# Is Co-production Just a Pipe Dream for Applied Health Research Commissioning? An Exploratory Literature Review

**DOI:** 10.3389/fsoc.2019.00050

**Published:** 2019-06-24

**Authors:** Doreen Tembo, Elizabeth Morrow, Louise Worswick, Debby Lennard

**Affiliations:** ^1^Wessex Institute, University of Southampton, Southampton, United Kingdom; ^2^Independent Researcher, Research Support Northern Ireland, Killyleagh, Ireland; ^3^NHS England, Taunton, United Kingdom; ^4^Public Member of National Institute for Health Research Evaluation Trials and Studies Coordinating Centre Patient and Public Involvement Reference Group, University of Southampton, Southampton, United Kingdom

**Keywords:** patient and public involvement, public engagement, co-creation of knowledge, co-production, research commissioning, research priority setting, citizen participation, biomedical

## Abstract

**Background and Rationale:** Internationally, the idea of “co-production' has become more popular in health research because of the promise of partnership between researchers and patients to create research that focuses on patients' needs. Patient and public involvement (PPI) at an early stage in deciding what research should be funded, can improve the quality and impact of research. However, professional power over the process places limits on the public practising their participatory rights for involvement in commissioning research that affects them and can leave members of the public feeling unheard or excluded, particularly within the context of early phase applied health research.

**Aim:** This article explores whether and how the public can be involved in the co-production of research commissioning early on in the process, with a focus on the power relations that pervade basic and early phase translational applied health research.

**Methods:** An exploratory literature review of international peer-reviewed and gray health research literature using structured searches of electronic databases and key search terms.

**Results:** There is very little literature that critically evaluates how PPI is embedded into the early phases of the commissioning process. The field of basic or early translational applied research appear to be particularly challenging. Four themes which emerged from the review are: reasons for PPI in research commissioning; benefits of PPI at strategic levels of research commissioning; contributions of patients and members of the public; improving PPI in research commissioning.

**Conclusion:** Although the public are being consulted at some stages of the research commissioning process, it is evident that the process of determining research priorities and agendas is far from being widely co-produced. Moving PPI from a consultative paternalistic model to a collaborative partnership model should be a priority for commissioners. Significant changes to communication, practices, systems, structures, or cultures that exclude patients and the public from contributing in meaningful ways, are needed to fulfill the potential of co-produced models of research commissioning.

## Introduction

### The Promise of Co-production

Internationally, the idea of “co-production” has become more popular in health research because of the promise of partnership between researchers and patients to create research that focuses on patient's needs. Patient and Public Involvement (PPI) at an early stage in deciding what research should be funded can improve the quality and impact of research. However, internationally there are very few examples of research commissioners involving patients or the public in decisions about research. This can leave members of the public feeling unheard or excluded by professionals.

Research commissioning is the most important stage of the research process for patients and the public to be involved as it gives the greatest potential to shape research agendas and to influence research funding (Oliver, 1996). However, research on decision making about future research priorities shows this rarely involves patients or the public. Decisions are more often made on the basis that technical rationalization of what research should be done, is more applicable than what is important to end users of research outputs.

Internationally in health services research PPI is widely recognized as being essential to the development of quality health services that are fit for purpose (Minogue and Girdlestone, 2010). Compared to health service delivery, PPI in health research management is globally a more recent movement and set of practices (Abrahams et al., 2004; Elberse et al., 2012; Gagnon et al., 2014; NIHR, 2015; PCORI, 2018).

Involving patients and the public in research, and especially in the early phases of research commissioning, such as research question or topic identification, priority setting, prioritization, and developing calls or advertisements for funding is thought to be crucial to overcome differential priorities between research funders, pharmaceutical companies and researchers, and the priorities of clinicians, patients and the public (Caron-Flinterman et al., 2005; Crowe et al., 2015). The consequences, as Chalmers and Glasziou (2009) describe of poor involvement of relevant stakeholders such as clinicians and patients in priority setting is an estimated avoidable waste of 85 per cent of global health research funding (Minogue et al., 2018).

### Defining PPI and Co-production

The history of involving the public in service provision in the UK, one of the earliest adopters of PPI, was catalyzed by the rise in consumerist thinking in the 1960s and 1970s, and democratic or rights-based approaches that arose thereafter (Ridley et al., 2002). Under the UK Health and Social Care Act 2001 publicly-funded organizations have a duty to involve the public in the planning and provision of health services.

In the UK in 2006 the National Institute for Health Research (NIHR) was established with a mandate to involve patients and the public in commissioning and delivering publicly-funded applied health research. The organization “consumers in research” now known as INVOLVE, a national advisory group for PPI, also joined the NIHR in the same year. A legacy of this organization is its widely used definition of PPI, which we utilize in this paper:

“*Research being carried out* “*with*” *or* “*by*” *members of the public rather than* “*to*”, “*about*” *or* “*for*” *them”* (INVOLVE^1^).

There is variation internationally in definitions, models and ways of thinking about PPI. There is for example no agreed nomenclature with *participation, engagement* and *involvement* often being used interchangeably. There is also great variation, dependant on the country's historical development of democracy, in the mix of institutionalized vs. contestory forms of involvement in healthcare (Slutsky et al., 2016). Within the UK context, involvement within health research funding tends be embedded within institutionalized mechanisms and processes.

Theoretically there are different levels at which people can be involved, as highlighted in Hogg's (1999) models of involvement in service development which closely relate to the INVOLVE levels of involvement in research (*consultation, collaboration, user-led and co-production*). *Paternalistic models* of involvement, assume that professionals know best, and hence lend themselves to involvement at the consultative level. The *Partnership models* of involvement lend themselves more to collaborative approaches to involvement. The *Consumerist model* describes consumers in charge or user-driven or controlled involvement. Finally the *Autonomy model* emphasizes the importance of valuing individuals and the different perspectives patients and professionals bring, and is closely aligned with involvement at the co-produced level as defined by INVOLVE (Hickey et al., 2018).

Co-produced research harnesses the principles of sharing of power, including all perspectives and skills, respecting values and the knowledge of all those working together on the research, reciprocity and building and maintaining relationships. However, this understanding of co-production, while acknowledged to be valuable, has been criticized as being idealistic given current cultural, institutional and regulatory constraints (Madden and Speed, 2017; Green and Johns, 2019; Paylor and McKevitt, 2019).

### Previous Research

The evidence base for PPI, and especially effective co-produced approaches in the early phases of research commissioning is underdeveloped (Nilsen et al., 2006; Oliver et al., 2008), especially when compared to PPI elsewhere in research (Shippee et al., 2015) or health services commissioning (Sheaff et al., 2015). A rapid review carried out by Manafò et al. (2018), which we include in this review, utilized rapid review methodology to explore existing evidence around the different approaches that could be utilized to enable PPI in priority setting in health ecosystems and health research. There is a need to further explore these and other different approaches and mechanisms, and the influence and impact PPI might have in the early phases of the research commissioning context (Staniszewska et al., 2011). This information could inform innovative collaborative and co-produced approaches which maximize the benefits of PPI through the research commissioning process.

PPI is perceived to be particularly challenging in the commissioning of clinical research, which might not have direct relevance to human health or patient outcomes, due to its early placement in the applied health research translational pathway (Caron-Flinterman et al., 2005; Dobbs and Whittaker, 2006). Some researchers might assume that patients may be put off engaging in such research due to finding science boring, irrelevant, or intimidating (Dobbs and Whittaker, 2006). Other researchers may be apprehensive because PPI can mean a different way of working that challenges established notions of professionalism (Thompson et al., 2009).

Concerns about tokenism and meaningful PPI are found throughout the research literature but are used as a catchall term that may not fully convey the limiting forces of professional power. Tokenism can be defined as the policy or practice of making only a symbolic effort to involve people (Domecq et al., 2014) or failure to develop approaches that enable people to contribute in meaningful ways (Supple et al., 2015). Unequal power relations between experts and the public can be challenging for both parties, and co-production and power sharing may be an unfulfilled ideological goal.

### Aims of the Review

The aim of this exploratory literature review was to draw on international health research literature to explore some of the contextual complexities and the potential challenges of PPI in the early stages of research commissioning, with a particular focus on early translational applied health research.

The questions we explored were (a) whether and how the public can be involved in the co-production of knowledge in research commissioning? (b) What are the specific challenges in the context of basic and early phase translational applied health research? The paper draws on the findings of the exploratory literature review to address these questions.

We used the notion of co-production to consider how research might overcome differentials in power between professional and public members, which may limit meaningful PPI. Drawing on examples and findings from the literature, in the discussion, we suggest possible ways forward for innovation and improvement of meaningful PPI.

Our focus is the potentially challenging field of commissioning early phase applied health research because it is here that commissioning is far less likely to involve PPI than in the later phases of the “bench-to-bedside' research process (Callard et al., 2012). The reasons for which we will also explore.

## Methods

### Approach

**Figure 1:**
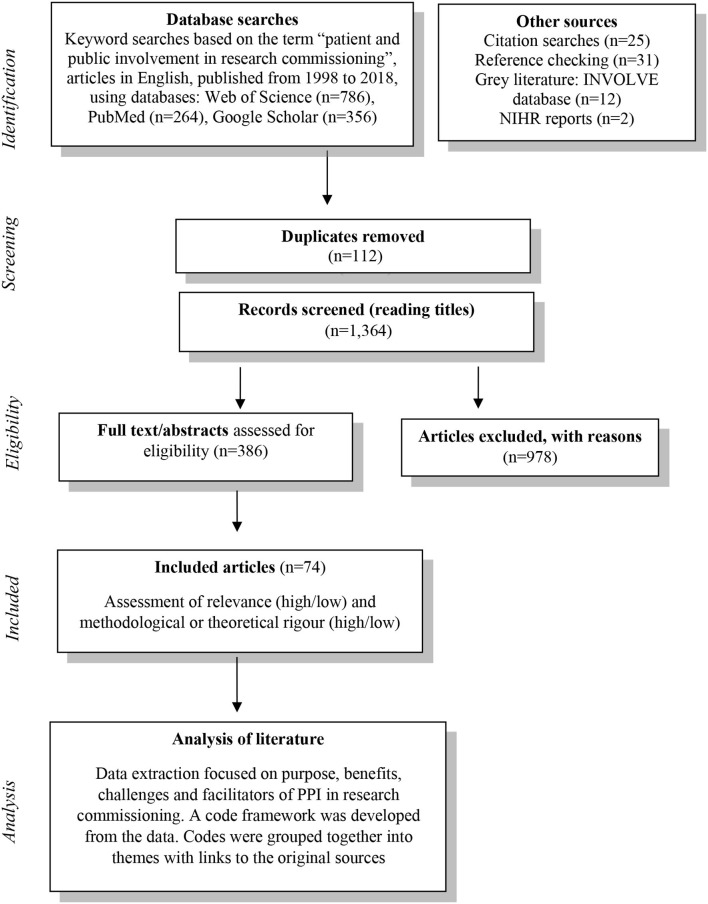
Flow chart of exploratory review method.

An exploratory literature review was carried out between May and August 2018. Owing to the disparate and scarce nature of evidence on PPI in research commissioning, a systematic review was unlikely to yield useful results that can inform practice. Therefore, an exploratory approach was chosen to seek out relevant published literature to allow us to consider the issues and challenges of PPI in research commissioning. The method is illustrated by Figure 1.

We sought information about how to enable meaningful and effective approaches to involvement, as well as clarification about the meaning of tokenistic PPI in this context. We were interested in learning about ways of working that enable patients/public representatives to contribute to decision-making processes and the types of impact that PPI can have. The study team included two public contributors who were consulted throughout study.

### Inclusion/Exclusions

The review explored issues about PPI in the commissioning of health research, including health services, health care, public health, clinical, and biomedical research. Included articles were those that addressed issues about: (i) any type of patients and public groups involved and their roles e.g., public reviewers, patient representatives or lay members, (ii) contexts of involvement in stages of the commissioning process, (iii) approaches to involvement, for example commenting on commissioning materials or involvement in face-to-face meetings, informing decisions, or shared decision-making practices, (iv) evidence of influence or impact of involvement on commissioning decisions, practices, or outcomes.

We sought journal articles (including empirical studies and literature reviews) and gray literature (including reports, discussion papers, commentary, and opinion pieces) where these offered useful insights and learning and were published in the English language.

Due to the limitations of time and resources we excluded articles published in other languages. We excluded articles that did not relate to health research commissioning, for example PPI in commissioning social care research or health professional education.

### Search Strategy

The search strategy was to identify relevant evidence and information using:

- web-based searches of Web of Science, Google Scholar and PubMed to search the international scholarly literature; explore related works, citations, authors, and publications; and the retrieval of documents through online libraries or on the web.
- searches of the INVOLVE Evidence Library for gray literature e.g., PhD studies, organizational reports, and bibliographies.
- searches for NIHR unpublished reports and documents relating to PPI in commissioning.

### Key Search Terms

Searches used the key term “patient and public involvement in research commissioning' and variations on the term (e.g., patient involvement in funding agencies). A comprehensive search drew on the search terms used by Brett et al. (2014) in their systematic review of the impact of PPI. It combined sets of terms including and relating to patient and public involvement (consumer, citizen, client, carer, lay, service users, survivor, stakeholder, family, relative); type of involvement (particp^*^, collaborat^*^, engage^*^, partner^*^, consult^*^, evaluat^*^) and commissioning (funding agencies, research briefs, research funding, identifying research priorities, research priority setting, scoping review). MeSH terms were used to expand the searches (patients, public, economics, research, funding).

### Data Extraction

Identified articles deemed to be relevant to the aim of the review were retrieved in full for analysis. Data were extracted into themed categories in Microsoft Word and key data extracted included the following: the author; the year and country; the aims or focus of the article; the methods used for PPI; the type of patients or groups of the public involved; key issues, findings or implications.

### Analysis

The approach to the analysis was to explore and identify themes in the data (Denzin and Lincoln, 2005) reflecting the aims of the review to explore some of the contextual complexities and the potential challenges of PPI in the early stages of research commissioning. We read each article and considered the main issues raised in relation to the questions of whether and how the public can be involved and specific challenges associated with involvement in the commissioning context. As issues were identified, these were given a code (a title phrase or word representing the issue), and in this way a code framework was developed from the data to indicate patterns across the data (Braun and Clarke, 2006). Codes were grouped together into emerging themes (purpose, benefits, challenges, facilitators) with links to the original sources (Denzin and Lincoln, 2005). In the analysis the notion of co-production was used as a lens through which to consider issues of power (Hickey et al., 2018) between professionals and public members. For example we looked for examples of power sharing in the data, e.g., new roles and responsibilities of PPI members, evidence of shared decision-making, and approaches to supporting positive interactions and communication. Tables were used to present synthesized themes and links to original sources.

### Rigor

A study protocol for the review was developed and revised by team members, including identification of databases to be searched and key search terms. Strategies for minimizing biases in the search strategy were as follows. (a) One team member independently cross-checked a sample of 20 returned papers against included/exclusion criteria. (b) Members of the team discussed and reached agreement on the importance of emerging themes in the analysis. (c) Inclusion and use of gray literature to extend the searches beyond peer reviewed articles.

## Results

The review identified 74 relevant papers, reports and articles about PPI in health research commissioning. The results of the review confirmed the lack of published material specifically around PPI in the early phases of the commissioning processes of early phase applied health or basic health research. The review did yield results on PPI in commissioning of applied health research that was further along the translational pathway. Here we present summary results of the main findings with some representative references to the body of literature from the review.

The structure of the results is presented according to four themes that emerged:

- Reasons for PPI in research commissioning
- Benefits of PPI at strategic levels of research commissioning
- Contributions of patients and members of the public
- Improving PPI in research commissioning.

### Reasons for PPI in Research Commissioning

The review demonstrated that PPI in research commissioning predominantly operated within a paternalistic model, with public members being consulted rather than more inclusively involved in the commissioning processes as co-creators of knowledge and co-producers of commissioning decisions and processes. Reasons for PPI were rarely given or explained, which could reflect the fact that PPI is often a requirement of being awarded central funding in the UK context. However, this is not the case in other countries or for all health research that is funded by other means.

### Benefits of PPI at Strategic Levels of Research Commissioning

Despite operating within a paternalistic environment, several benefits to involving patients and the public, beyond getting them to provide views about priorities for research, were identified in the literature. These are summarized in Table 1 and include research priorities becoming more relevant to users; broader perspectives being brought into commissioning decisions; research being more likely to be ethical, inclusive and fair; the contribution of public contributors' skills and knowledge to commissioning decisions; and encouragement of PPI in funded research.

**Table 1:** Benefits of PPI in research commissioning.

**Possible benefits**	**Ways PPI influences commissioning**
Research priorities are more relevant to users	• PPI members are likely to ask how the research will benefit patients (Brett et al., 2014; Domecq et al., 2014; Shippee et al., 2015) • PPI in developing the focus and aims of research can mean it is more likely to meet the needs of patients (Rhodes et al., 2002; O'Donnell and Entwistle, 2004; Abma, 2005; Caron-Flinterman et al., 2005; Hewlett et al., 2006; Howe et al., 2006; Nilsen et al., 2006; Lindenmeyer et al., 2007; Shah and Robinson, 2007; Gagnon et al., 2011) • Research questions or hypotheses can be developed to focus on issues that are important to beneficiaries (McCormick et al., 2004; O'Donnell and Entwistle, 2004; Viswanathan et al., 2004; Abma, 2005; Hailey and Nordwall, 2006; Howe et al., 2006) • Poor research ideas are abandoned (Boote et al., 2014)
Broader perspectives are brought to commissioning decisions	• Patients or members of the public may contribute experiential knowledge, which can corroborate or enhance scientific or professional knowledge; (Andejeski et al., 2002; Oliver et al., 2009) • Experiential knowledge can enhance the research brief through co-production or co-design of solutions (Crowe et al., 2015; Manikam et al., 2017)
Research is more likely to be ethical, inclusive and fair	• PPI members are likely to ask whether the research is ethical or moral (Morgan et al., 2005; Staley, 2009; Brett et al., 2014; van Bekkum et al., 2016) • PPI can enhance research practices such as ethical recruitment (Oliver et al., 2009; NIHR CLAHRC, 2017) and transparency (Hutchison et al., 2017) • Researchers develop skills and knowledge in partnership working (Brett et al., 2014; Gagnon et al., 2014) • Involving members of the public can encourage interdisciplinarity (Oliver and Gray, 2006)
PPI members contribute skills and knowledge to commissioning decisions	• Patients and members of the public bring personal assets to commissioning processes, such as skills, abilities and links to charities or community organizations (Coulter, 2004; Abma, 2018) • Patients and members of the public provide time and support e.g., comments that lead to clearer briefs (Brett et al., 2014)
PPI in commissioning encourages PPI in research	• People can benefit from their involvement and be more likely to engage in research or civic activities in the future (Fudge et al., 2007) • PPI can support access to community networks and wider groups of the public or disseminate information (Brett et al., 2014; Crowe et al., 2015; Manikam et al., 2017; Simpson et al., 2018)
Commissioning processes are more transparent and accountable	• The public oversee research and are given access to research information (Greenhalgh et al., 2017) • Research organizations are publicly accountable (Resnik, 2001)

### Contributions of PPI Members

The review also highlighted specific activities and contributions patients and public members make to the overall commissioning process. These have been summarized in Table 2 and include identifying topics, prioritizing topics, assessment, review of evidence, synthesizing results, and writing research briefs.

**Table 2:** Contributions of PPI members to research commissioning.

**Activities**	**Examples of PPI roles/contributions**
Identifying topics	• Inviting members of the public to suggest an issue, condition or problem that research could help to address (Oliver et al., 2004; Royle and Oliver, 2004; Menon and Stafinski, 2011; PCORI, 2018) • Responding to an organizational survey or review of future challenges or participating in exercises to identify needs for future research (Moran and Davidson, 2011; Franck et al., 2018) • Patients with a common interest raising issues or bringing issues to the attention of the research community through their engagement with health services or patient networks (Morris et al., 2011; Brady and Preston, 2017)
Prioritizing topics	• Convened groups (e.g., focus groups) of patients participating in activities to vote for, or rank, priority areas (Husereau et al., 2010; Gagnon et al., 2014; Pittens et al., 2014; Rikkers et al., 2015; NIHR CLAHRC, 2017; Parsons et al., 2017; Rawson et al., 2018; Truitt et al., 2018) • Consensus exercises or dialogue on research priorities (Smith et al., 2005; Abma, 2018) • Patient groups or voluntary organizations putting forward a case for research into topics that are felt to be important (JLA, 2018) • Contributing to developing or implementing a commissioning body's research strategy (Oliver and Gray, 2006; Moran and Davidson, 2011; Gamble et al., 2014; NIHR, 2015)
Assessment	• PPI members of research advisory panels and boards (Entwistle and O'Donnell, 2003; Oliver and Gray, 2006) • Expert patients and/or carers with direct experience of a health condition or illness providing comments on the value of research from a patient's perspective (Brett et al., 2014) • Representatives of patient groups or organizations advising on the feasibility of patient participation in research studies (Crocker et al., 2017)
Review of evidence	• Scoping the field for existing evidence involving patients and the public in identifying evidence or to identify needs (Smith et al., 2008; Oliver et al., 2009; Bragge et al., 2011) • Reports of research undertaken by voluntary organizations or patient groups which are fed into a review (Abma, 2018) • Evidence generated through focus groups, citizens juries (Entwistle et al., 2008; Gooberman-Hill et al., 2008) or action research (Greenhalgh et al., 2017)
Synthesizing results	• Public reviewers pointing out where there might be gaps in understanding (NIHR BRCU, 2017) • Raising patient perspectives of what is important to know (Caron-Flinterman et al., 2005; Crocker et al., 2017; JLA, 2018) • Patient reviewers contributing to committee meetings about research briefs (NIHR BRCU, 2017)
Writing research briefs	• Contributing to specifying the focus of research briefs (Oliver et al., 2004) • Commenting on draft research briefs (Brett et al., 2014) • Reviewing research briefs (NIHR BRCU, 2017) • Reviewing plain English summaries of briefs (Oliver et al., 2009)

### Improving PPI in Research Commissioning

The review discovered that new priority setting projects are being developed around the world (in the UK, US, Australia, Netherlands, and Canada) to build partnerships between patients and professionals (Bragge et al., 2011; Gagnon et al., 2014; Tong et al., 2015; Pratt et al., 2016; Ghisoni et al., 2017; Abma, 2018; JLA, 2018; Manafò et al., 2018) (see Table 3).

**Table 3:** Priority setting approaches that involve patients and the public.

**Model and setting**	**Model description**
James Lind Alliance Priority Setting Partnerships (UK)	• Priority Setting Partnerships (PSPs) enable clinicians, patients and carers to work together to identify and prioritize uncertainties about the effects of treatments that could be answered by research. PSPs identify treatment uncertainties (questions about treatments which cannot be answered by existing research) which are important to all groups (often a Top 10 list) of jointly agreed priorities which are publicized widely (JLA, 2018)
Dialogue Model for research agenda-setting (Netherlands)	• The Dialogue Model actively engages patients in research agenda setting to balance power. It provides guidelines to develop a shared research agenda among patients and other stakeholders. The approach involves phases of exploration, consultation, prioritization, integration, programming, implementation (Abma, 2018)
Global Evidence Mapping (Australia)	• Evidence mapping describes the quantity, design and characteristics of research in broad topic areas, in contrast to systematic reviews, which usually address narrowly-focused research questions. The breadth of evidence mapping helps to identify evidence gaps and may guide future research efforts (Bragge et al., 2011)
Deep Inclusion Method/CHoosing All Together (US)	• This model consists of three dimensions: breadth, qualitative equality, and high-quality non-elite participation. Deep inclusion is captured not only by who is invited to join a decision-making process but also by how they are involved and at what point in the process non-elite stakeholders are involved (Pratt et al., 2016)
Health Technology Assessment conceptual framework for patient involvement (Canada)	• Patients and their representatives are involved in activities to identify potential HTA topics, review vignettes or research briefs developed to inform the prioritization of topics, participate in deliberation sessions for prioritizing HTA topics, and develop the assessment plan of the topic prioritized (Gagnon et al., 2014)

Manafò et al. (2018) review of these priority-setting approaches concluded they are inclusive and objectively based, while being specific to the priorities of stakeholders engaged in the process. Key limitations identified were a lack of evaluation data on the success and extent to which patients were engaged, issues pertaining to feasibility of stakeholder engagement, coordination, communication, and limited resources.

Evaluation of nine projects that used the Dialogue Model (Abma et al., 2015) found patient involvement in agenda-setting is not automatically followed by patient involvement in programming and implementation. The authors recommend that support is needed during the process to organize patient involvement and adapt organizational structures like review procedures. Facilitating factors for success of the model include the importance of ownership; the value of dialogue for personal and mutual understanding; relational empowerment and critical awareness raising among patients; the importance of responsibility, responsiveness and trust; support in working with co-researchers; and the issue of representation (Abma, 2018).

Gagnon and colleagues of the Canadian Health Technology Assessment (HTA) programme have generated a conceptual framework for interventions to promote patient involvement in the early stages of HTA (Gagnon et al., 2014). Outcomes of PPI are evaluated with patients and their representatives using interviews and observations. These priority-setting projects and activities are promising but more needs to be done to test them out in different research funding contexts and particularly in early translational applied health research commissioning.

The review found examples of ways to facilitate PPI in commissioning, which could be utilized for the identification and prioritization stages of the process within early stage applied health research. In summary, these are:

- Planning for meaningful involvement all the way through the commissioning process (Oliver et al., 2004, 2009)
- Finding ways to expand opportunities for wider and effective participation and engagement with the public (Willis, 1995; Abelson et al., 2003; Oliver et al., 2008; INVOLVE, 2012; Morrow et al., 2013; Rikkers et al., 2015; Franck et al., 2018; Rawson et al., 2018; Simpson et al., 2018; Truitt et al., 2018)
- Building positive attitudes toward PPI as well as positive relations between stakeholders (Pittens et al., 2014; Abma et al., 2015; Abma, 2018). This could be facilitated by developing guidance, training and support for patient and the public contributors, Chairs of commissioning bodies and teams, including opportunities for shared learning (Boote et al., 2002; Caron-Flinterman et al., 2005; Oliver et al., 2008; INVOLVE, 2012)
- Encouraging organizations to assess the quality and impact of public involvement in commissioning (Oliver et al., 2015)
- Supporting commissioning teams to assess and provide feedback about processes and outcomes (O'Donnell and Entwistle, 2004; Howe et al., 2017).

## Discussion

### Variation in Opportunities for PPI in Commissioning

The review reveals a story of PPI opportunities for involvement in commissioning that ranges from ineffectual tokenism to meaningful co-creation of knowledge. Our findings suggest that while some research funders are fully committed to PPI at every stage, others have not given sufficient consideration to the benefits of PPI identified in this review. Indeed our findings do little to contest previous observations that commissioners may be concerned that PPI will distort research agendas (O'Donnell and Entwistle, 2004). Improved utilization of the review identified activities that patient and public representatives can be involved in by research funders would move the involvement model and levels from one of paternalism and consultation to one that is partnership-based and collaborative.

Most research funding organizations are open to asking patients to submit their views about priorities for research (e.g., a website where people can make suggestions for research), and some organizations go out and engage patients and groups of the public about their views about research needs. While the review highlighted novel and effective approaches to priority setting that include patients and the public, it also demonstrated that there is relatively little evidence, beyond identification and prioritization of research topics (e.g., James Lind Alliance Priority Setting Partnerships), of wide-spread co-production or co-creation in the development of prioritized research areas and funding calls.

The study by van Bekkum et al. (2016) which looked at ten UK agencies that fund health or medical research found involvement was not routinely incorporated into the planning of funding calls and there was little evidence of PPI being driven by democratic imperatives or rights-based arguments. Agencies and commissioning groups working within specific areas of health and medicine tend to promote particular definitions and practices which determine the boundaries in which researchers in these areas understand and practice PPI (van Bekkum et al., 2016). Professionals may be generally in favor of PPI but may believe that ultimately decisions about which research gets funded should be made by the professionals who are held accountable for these decisions (Oliver et al., 2004).

There are some strong examples of how the public can be involved in the co-production of knowledge in research commissioning. For example, some UK research funders, such as the NIHR and Medical Research Council, and US funders such as the Patient Centered Outcomes Research Institute, have research management frameworks for PPI which may include patients and members of the public being asked to review documentation that support prioritization of research topics or act as members of research prioritization committees (Oliver et al., 2009). However, even within this framework, it appears that some commissioning activities (e.g., defining assessment criteria, reviewing evidence, synthesizing results, writing documents for the consideration by committees, and funding decisions) may be undertaken by professionals without public input. Power is therefore balanced more toward researchers and funding organization staff than patients and public representatives. This is often the case for basic and the early applied health research commissioning context.

### The Effects of Power Differentials

The review did not identify literature that focused on early stage commissioning processes for basic or early phase applied health research. The literature reveals some of the specific challenges in the context of basic and early phase translational applied health research. Perhaps most significant, is that professional skepticism and resistance manifest in subtle yet powerful ways that can limit co-production to a pipe dream (Chase et al., 2000). Even though the usefulness of patients' experiential knowledge alongside professional and clinical knowledge is widely accepted (Boote et al., 2002; Brett et al., 2014), it can be less clear how to integrate this type of knowledge into decision-making (Caron-Flinterman et al., 2005), to share ownership of decisions, and to assess decision-making effectiveness (Entwistle and O'Donnell, 2003). Researchers and funders may therefore employ tokenistic PPI, especially within the UK context where PPI is either increasingly encouraged or mandated.

The literature indicates that tokenism can be caused by lack of awareness or resistance to involvement amongst professionals, but can also be caused by practices, systems, structures or cultures that exclude patients and the public from contributing in meaningful ways (Supple et al., 2015). The technical nature of early phase translational research and the bureaucratic nature of commissioning may be a reason why the public are excluded from some commissioning activities. However, the literature demonstrates that public contributors' understanding of the technical clinical subjects, the language and science are not a necessary barrier to involvement.

When investigating patient and public involvement in biomedical research, Caron-Flinterman et al. (2005) asserted that training may support patients and the public to understand highly scientific or technical research. Further widespread use of non-technical language by professionals and plain English summaries may better enable involvement. Training for commissioning teams could cover inclusion strategies in patient–expert partnerships thereby enabling a better platform for both parties to effectively communicate and contribute to collaborative or co-produced approaches (Elberse et al., 2011).

### Areas for Innovation and Improvement

Commissioning research requires informed judgements to be made about what research is important, and could lead to potentially significant results and impactful outcomes (Oliver et al., 2009). A sole focus on PPI as a participatory right endangers the involvement process into becoming a tokenistic activity that is consultative at best. Previous discursive papers on PPI suggests three different lines of thinking about the reasons for PPI in commissioning. These are: moral (to assure participative rights to involvement) (Boote et al., 2002; Coulter, 2004), methodological (to improve the quality and relevance of research to society) (Fisher, 2002; Chalmers and Glasziou, 2009), and impact (health, political, legislative, economic and societal impact). Moral or rights-based arguments suggest that PPI should be integral to research from the earliest stages as an intrinsic participatory right (Boote et al., 2002; Coulter, 2004). Methodological and impact based motivations, on the other hand, do not necessarily recommend involvement through the whole processes where it does not add value. It remains imperative that commissioners embed the moral or participatory rights-based driver as a key underlying factor that propels involvement in the system. Additionally an effective commissioning system must also consider and harness the methodological and impact drivers and benefits of PPI, such as those identified in this review, to create buy-in from all stakeholders.

Increasingly commissioning bodies are recognizing that the issue of what constitutes a rational discourse for future research, is a complex interplay of issues about how principles of patient need and rights translate into research contexts. Arguments against PPI warn against the lack of objectivity, possible bias, and individual self-interest of members of the public when it comes to making decisions about the allocation of research funds. Notions of the rights of the public to participate in all areas of health care—captured in the phrase “nothing about us without us”—are undermined by the apparent irrationality of involving members of the public in rational decisions about the allocation of research funds based on gaps in the evidence base and the feasibility, methods, and merit of the science in question.

Preoccupation with representation issues and concerns about the professionalization of lay members has directed too much attention to questions about the effectiveness of individual PPI representatives. Instead, PPI could be improved by examining the presuppositions and validity dimensions of everyday communication (normalized discourse) between professionals and PPI members. In relation to PPI in research commissioning this could include using reflective studies, to activate reflection on the unease, tensions and concerns about tokenism.

Improving opportunities for PPI requires the provision of meaningful spaces for dialogue, exchange and decision-making that suit different types of professionals and PPI representatives, as well as the public more generally. Early explicit exploration of different PPI roles and contributions with members of the public may assist effective participation and satisfaction. Singular PPI models are unable to effectively respond to the pluralism in experiences, values and opinions that different members of society hold.

Much could be gained from the involvement of third sector groups with local, regional or sector-wide views. Other approaches could be e-consultation or crowd-sourcing research topics and prioritizing them with a virtual public and professional community of practice, democratic prioritization (through voting), use of social media, or holding James Lind Alliance style priority-setting and consensus-building exercises to identify and prioritize areas of future focus (Rawson et al., 2018; Simpson et al., 2018; Truitt et al., 2018). In their review, Oliver et al. (2008) suggest a particularly fruitful method for involving the public in setting large-scale research agendas. The method was a combination of collaboration and consultation, with lay people taking leading roles in consulting peers in their networks.

There is a need for more innovative thinking about ways to relate to “seldom heard” and “hard to reach” populations, such as black and minority ethnic groups and persons with disabilities, by diversifying languages and mechanisms of communication. Creating mechanisms for engagement in commissioning that are more inclusive of diversity (e.g., by age, gender, ethnicity, socioeconomic background, and other characteristics) and reach out to wider groups of patients and the public (e.g., different experiences of health and illness, different patient groups, carers and those who are well) can help to stimulate interest and participation in commissioning. Combining different approaches can bring a more diverse range of people and their perspectives and views to the commissioning process that are more representative of diverse service user needs and priorities (Oliver et al., 2008).

More could be done to find ways to talk about complex technical ideas and research methods in accessible plain English (and to celebrate those professionals who find comprehensible expression), and to raise awareness of behavior that intimidates, side-lines or stigmatizes individuals. If we do not want PPI to be tokenistic in this area, it is important to develop policy, standards, guidance, roles, training, information, communication technologies and digital platforms (e.g., websites and social media) to support patient and public involvement in different research commissioning activities.

It is vital for people who find themselves occupying positions of power in the commissioning system to turn a critical eye toward the system. Research areas that appear to be far removed from immediate patient benefit due to being positioned early in the applied research translational pathway, especially need to better engage the public. Those in power should seek to show how the system is responsive to societal needs, for example showing the impact of commissioned research on patients or other beneficiaries (Pramesh et al., 2016). Therefore, a key issue for funders going forward is how to build capacity to adapt and absorb change brought about through co-production and the co-creation of new ways of commissioning.

### Limitations

This review does not cover some of the practical challenges of PPI funders may face, including access and issues of reimbursement and payment. These issues, in different contexts, have been explored elsewhere in the literature and guidance to overcome some of these challenges is available from INVOLVE (Snape et al., 2014). The main limitation of the review is the focus on professionally defined commissioning approaches and models. It does not include lay groups taking the initiative through user-led research, or commissioning practices of user-led research organizations.

Limitations of the literature reviewed are the deficit of high-quality research studies (no trials were identified), the reliance on literature reviews, and small-scale evaluation studies carried out on single units or programmes. While international literature was included, differences in language and terminology of involvement, engagement and participation between countries are a limitation of the searches. Including languages other than English would have reduced bias but this was not possible within the limited resources for the review.

## Conclusions

Although the public are involved in some countries, at some stages of the research commission process, it is clear that the process of agreeing research priorities is a long way from being co-produced and can be tokenistic. Tokenism can be caused by lack of awareness or resistance to involvement amongst professionals, but it can also be caused by highly structured commissioning systems, technically defined subject areas, and tasks that may exclude patients and the public from contributing in meaningful ways.

Addressing concerns about tokenism requires commissioners to critically reflect on current PPI practices and to devise ways of working that are meaningful and worthwhile for everyone involved. PPI could change from a minimal and minor role to a true partnership role, if improvements were made to communication, practices, systems, structures and cultures that stop patients and the public from contributing in meaningful ways.

If we want to avoid tokenism in PPI, it is important that commissioning organizations develop mechanisms to enable commission teams to secure the involvement of patients and the public through a range of options for engagement and involvement, including use of face-to-face methods and digital platforms. New, more distributed approaches to commissioning could be based on collaboration or partnership models, which bring together patients, carers and clinicians to create truly co-produced research agendas.

## Data Availability

No datasets were generated or analyzed for this study.

## Author Contributions

All authors are part of the project team comprising the larger work programme from which this paper draws its data. All authors contributed to the development of the paper, with DT and EM taking primary leadership in drafting and co-authors (LW, DL) providing detailed feedback on drafts.

### Conflict of Interest Statement

The authors declare that the research was conducted in the absence of any commercial or financial relationships that could be construed as a potential conflict of interest.

## Footnotes

^1^INVOLVE. What is public involvement in research? Retrieved from: http://www.invo.org.uk/find-out-more/what-is-public-involvement-in-research-2/
